# Hacia un cribado personalizado del cáncer de próstata

**DOI:** 10.1515/almed-2019-0012

**Published:** 2020-01-07

**Authors:** Xavier Filella

**Affiliations:** Servicio de Bioquímica y Genética Molecular (CDB), IDIBAPS, Hospital Clínic, Barcelona, Cataluña, España

**Keywords:** antígeno específico de la próstata (PSA), cáncer de próstata, cribado, PHI, 4Kscore

## Abstract

La utilidad del antígeno específico de la próstata (PSA) en el cribado del cáncer de próstata (CaP) es una cuestión controvertida. Los resultados publicados son polémicos en cuanto a la consecución de una disminución de la mortalidad y al rigor metodológico. No obstante, hay coincidencia en observar la relación del cribado con un aumento del número de CaP indolentes.

La controversia no se limita a la incertidumbre que rodea la utilidad del cribado, sino también al intervalo de edad en que podría ser aplicado, la definición de grupos de riesgo según un PSA basal, el intervalo de las mediciones de PSA o la inclusión de otros biomarcadores (PHI, 4Kscore). Actualmente, la mayoría de las sociedades no aconseja la práctica generalizada del cribado, pero sí lo admiten tras un dialogo informativo con el paciente, evaluando beneficios y riesgos.

En esta reflexión deben valorarse tanto los riesgos de hacer el cribado (falta de especificidad del PSA, sobrediagnostico) como los riesgos asociados a no hacer el cribado (aumento de pacientes que debutan con metástasis). Recientemente, diversos autores abogan por un cribado personalizado que podría cambiar el balance entre beneficios y riesgos y hacer oportuna, en función del riesgo de cada individuo, su implementación.

## Introducción

El cáncer de próstata (CaP) es el segundo tumor más frecuente en el sexo masculino en el mundo y el más frecuente en Europa [1]. Datos publicados recientemente indican que también en España es el tumor más frecuente en el sexo masculino, con 33.370 nuevos casos en el año 2015 [2]. Se ha observado, tanto en España como en otros países occidentales, un aumento en su incidencia en el curso de los últimos años, llegando a un máximo durante el año 2007, principalmente a causa de la introducción de la medida del antígeno específico de la próstata (PSA) en su detección. La medida de PSA en el diagnostico precoz del CaP es una práctica controvertida, dadas las limitaciones del PSA, tanto desde la perspectiva de su especificidad como en lo que refiere al sobrediagnóstico y sobretratamiento de la enfermedad, dado que se detectan numerosos tumores indolentes, de crecimiento lento y asintomático. A causa de ello, actualmente, las guías de práctica clínica no recomiendan de forma generalizada el empleo de PSA en el cribado del CaP, si bien la mayoría no lo excluye tras haber informado minuciosamente a los pacientes de los posibles beneficios y perjuicios de dicha práctica.

## Estudios de cribado

Los datos actualmente disponibles sobre la utilidad del PSA en el cribado del CaP han sido aportados por dos amplios estudios de cribado llevados a cabo en Europa y Estados Unidos. El estudio europeo, denominado *European Randomized Study of Screening for Prostate Cancer* (ERSPC), incluye 162.387 hombres de 7 países europeos, mientras que el estudio norteamericano, denominado *Prostate, lung, colorectal, and ovarian screening trial* (PLCO), incluye 76.693 hombres. Los resultados obtenidos por parte de ambos estudios han sido contrapuestos. El estudio europeo, según datos publicados en 2011, obtuvo, tras un seguimiento medio de 9 años, una disminución del 20% en la mortalidad en el grupo en que se realiza el cribado [3], mientras que el estudio norteamericano mostró que la realización del cribado no ofrece ningún beneficio tras realizar un seguimiento de 7–10 años [4]. En 2012 se publicaron resultados actualizados de ambos estudios confirmando con un mayor tiempo de seguimiento los resultados opuestos previamente publicados [5], [6]. Los resultados positivos del estudio europeo han sido ratificados ulteriormente con tiempos medios de seguimiento de 13 y 16 años [7], [8]. En cambio, los resultados recientemente publicados en un tercer estudio denominado *Cluster Randomized Trial of PSA Testing for Prostate Cancer* son negativos respecto a la utilidad del cribado con PSA [9]. Este estudio, realizado en el Reino Unido, incluye hombres de 50 a 69 años, en los cuáles, a diferencia de los estudios de ERSPC y PLCO, se realizó una única medida de PSA. El trabajo además, pese a que incluye 415.357 individuos, tiene algunas limitaciones adicionales, como un tiempo medio de seguimiento de únicamente 10 años y un cumplimiento de la biopsia cuando el cribado es positivo de tan solo el 40%.

Por otro lado, en su más reciente actualización, el grupo europeo subraya que el número de individuos que deben ser invitados a cribado para prevenir una muerte causada por CaP ha ido disminuyendo en relación al aumento del tiempo de seguimiento, siendo de 1947, 962, 742 y 570 individuos para tiempos de seguimiento de 9, 11, 13 y 16 años, respectivamente [8]. Igualmente, en sus dos últimas actualizaciones, el grupo europeo indica que el porcentaje de pacientes que son diagnosticados con metástasis o PSA superior a 100 μg/L era significativamente superior en el grupo control que en el grupo sometido a cribado [7], [8].

Tanto el estudio europeo como el norteamericano coinciden en observar un aumento en el número de casos de CaP diagnosticados en el grupo que realiza cribado con PSA, resaltando que ello comporta el sobrediagnóstico y sobretratamiento de un importante número de tumores con un bajo riesgo de progresión. Así, según datos del estudio europeo el porcentaje de tumores con un bajo riesgo de progresión fue del 56,4% en el grupo que hizo cribado y del 39,1% en el grupo control [8].

Así mismo, ambos estudios [3], [4] coinciden en mostrar que la reducción de mortalidad obtenida con el empleo del PSA en el cribado del CaP no se obtendría en ningún caso antes de los 10 años. Se justifica por la, en general, lenta evolución del CaP, con una amplia ventana de tratamiento en la mayoría de los casos. Por tanto, es desaconsejable la realización de cribado en individuos que tengan una esperanza de vida inferior a los 10 años, como coinciden en señalar las guías de práctica clínica.

La discusión de las diferencias entre ambos estudios [10] tampoco carece de interés, puesto que algunas diferencias en su diseño podrían explicarlas. Así, la contaminación con medidas de PSA en la rama control es muy superior en el estudio norteamericano (del 40 al 52%) que en el europeo (del 15 al 20%), lo que pondría en cuestión las conclusiones obtenidas por parte del estudio PLCO. Igualmente, las diferencias en el porcentaje de biopsias realizadas cuando el cribado fue positivo es radicalmente distinto: 86% en el estudio europeo y únicamente 35% en el estudio norteamericano.

Por otro lado, en contra del estudio europeo hay que señalar la heterogeneidad de resultados obtenidos en sus 7 centros, destacando los resultados mucho más favorables en la rama sueca del estudio, en la que se observó una reducción de la mortalidad cercana al 50% en el grupo de pacientes que han realzado cribado [11]. Contrasta con los resultados de la rama española del ERSPC, que, tras 15 años de seguimiento, no mostró diferencias en mortalidad en relación a la realización o no de cribado [12]. Las diferencias entre los distintos centros del ERSPC son atribuibles al diferente intervalo entre las rondas de cribado, al nivel de cumplimiento de las biopsias requeridas, al nivel de contaminación con determinaciones de PSA en el grupo control o al distinto tratamiento seguido en caso de diagnosticarse un tumor.

Recientemente, un estudio basado en un modelo de microsimulación señala que la disminución de la mortalidad debida a CaP se relaciona con el diseño del protocolo y su adecuado cumplimiento [13]. Los autores observan que la reducción de la mortalidad aumenta cuando el modelo incorpora una idealización de los parámetros previstos en el estudio, es decir, no contaminación con medidas de PSA en la rama control, riguroso seguimiento del protocolo, o cumplimiento de las recomendaciones de realización de una biopsia. En este caso, el estudio señala que la realización de cribado podría conseguir una reducción de la mortalidad por CaP de alrededor del 40%.

## Recepción de los resultados de los estudios de cribado

Diversas sociedades han elaborado sus recomendaciones sobre el cribado del CaP, teniendo preferentemente en cuenta los resultados de los estudios de ERSPC y PLCO. También ha tenido una gran influencia la publicación en 2011 de una revisión realizada por el *US Preventive Services Task Force* (USPSTF) en la que se desaconsejaba de manera contundente el cribado del CaP [14]. La revisión evaluaba los resultados de seis trabajos que consideraba metodológicamente bien planteados para concluir que el cribado del CaP reduce la mortalidad causada por esta enfermedad de forma mínima o inexistente, asociándose con una serie de prejuicios relacionados con la propia evaluación de los pacientes o su tratamiento que, además, subrayaba, en algunos casos podría ser innecesario. Esta conclusión quedaba reflejada en las recomendaciones que al año siguiente publicaba el USPSTF [15] que, teniendo en cuenta el balance entre daños y beneficios, eran contrarias al cribado del CaP basado en PSA para todos los grupos de edad.

Se abría en aquel momento una etapa en que las recomendaciones de las diversas sociedades se volvían más conservadoras y abogaban por reducir drásticamente el cribado del CaP mediante la medida de PSA. No obstante, otros autores han puesto de manifiesto que la práctica del cribado supone una reducción del número de pacientes que debutan con metástasis [7], [8], [16] y señalan que una consecuencia derivada del riesgo de no practicar el cribado podría ser un aumento del número de muertes causadas por CaP [17]. De hecho, el propio USPSTF en 2018 ha rectificado su valoración y recomienda, para hombres entre 55 y 69 años, llegar a una decisión compartida entre médico y paciente sobre la oportunidad del cribado, valorando sus riesgos y beneficios [18].

## Capacidad del PSA para predecir el desarrollo de un CaP a largo plazo

Diversos estudios muestran la capacidad del PSA para predecir el desarrollo de un CaP años o incluso décadas antes de ser diagnosticado. Esta observación sugiere que la liberación del PSA a la sangre sería un hecho temprano en el proceso de desarrollo del tumor. También indica que el PSA en pacientes jóvenes tiene una mayor especificidad de la que muestra en pacientes mayores, cuando los falsos positivos son numerosos, dada la elevada prevalencia de la hiperplasia benigna de próstata, enfermedad particularmente frecuente a partir de la sexta década de vida.

Los primeros datos en este sentido corresponden a un estudio finlandés que fue publicado en 1994 por Stenmann et al. [19]. En este estudio se diagnosticaron 44 CaP en una cohorte de 21.172 individuos de entre 45 y 84 años, que fueron enrolados entre 1968 y 1973. Los autores concluyeron que una elevación de PSA superior a 2,5 µg/L permitía predecir la posterior aparición de un CaP. Al año siguiente, Gann et al. [20], en un estudio que aumentaba el número de CaP hasta 366 casos, ratificaban estos resultados. Este estudio indicaba que, en comparación con los individuos con PSA menor a 1 µg/L, el riesgo relativo tanto de CaP como de CaP agresivo aumentaba conforme lo hacía la concentración de PSA.

Más adelante, dos nuevos estudios, mucho más amplios y con más tiempo de seguimiento, han corroborado la validez de PSA para predecir la futura aparición de un CaP. Loeb et al. [21], [22] indicaban que la medida de PSA en hombres de menos de 60 años permite estratificar el riesgo de ser diagnosticados en el futuro de un CaP. El estudio evaluaba una población de 26.000 varones que entre 1991 y 2001 participaron en un programa de cribado de CaP. Los autores subrayaban que el riesgo de que se diagnosticara CaP se incrementaba cuando el PSA era superior a la mediana poblacional, tanto para individuos de entre 40 y 49 años (con un aumento del riesgo de 14,6 veces) como para individuos de entre 50 y 59 años (con un aumento del riesgo de 7,6 veces).

Datos semejantes eran publicados por Lilja et al. [23] tras valorar, en un estudio caso-control, la capacidad predictiva del PSA en una población de 21.277 varones enrolados en un programa de estudio cardiovascular realizado entre 1974 y 1986. Para ello, los autores midieron PSA en 462 de los 498 individuos a los cuales se diagnosticó CaP, junto con 1222 controles apareados, que fueron seleccionados según la edad y la fecha de extracción de la muestra. Los autores observaban que el PSA medido entre los 44 y los 50 años permitía estratificar el riesgo de que se diagnosticara un CaP, el cual ascendía paulatinamente con la concentración de PSA. Así, la probabilidad de que fuera diagnosticado un CaP a largo plazo era del 4% si el PSA era menor a 0,51 µg/L, pero ascendía a 41% si era de 2,01 a 3 µg/L o superior al 60% cuando era mayor a 3 µg/L.

Resultados más recientes publicados por Vickers et al. [24] han mostrado que la concentración de PSA permite, además de predecir el futuro diagnóstico de CaP, estratificar el riesgo de tener a largo plazo un CaP metastásico o bien de morir a causa de esta enfermedad. El estudio evalúa 1167 varones de 60 años en los que se realizó una extracción de sangre en 1981 y que fueron seguidos hasta los 85 años. Los autores observaron que el 90% de las muertes debidas a CaP ocurrían en individuos con una concentración de PSA basal superior a 2 µg/L. Las consecuencias de estos datos son relevantes para establecer un programa de cribado del CaP. Una concentración basal, obtenida alrededor de los 60 años, permitiría decidir, para cada individuo, la necesidad o no de ser incluido en un programa de cribado. De hecho, los beneficios del cribado serían superiores en individuos con una concentración de PSA superior a 2 µg/L. En cambio, si la concentración de PSA es menor a 1 µg/L la probabilidad de poder padecer CaP es baja, e incluso en caso de que el tumor fuera diagnosticado, de forma muy poco probable pondría en peligro la vida del paciente.

## Sobrediagnostico y sobretratamiento del CaP

El sobrediagnóstico y sobretratamiento del CaP es uno de los retos más importantes que debe ser afrontado para establecer con éxito una estrategia de cribado de esta enfermedad. De hecho, los tumores con un bajo riesgo de progresión son un porcentaje importante de los nuevos diagnósticos de CaP. Así, según datos del ERSPC, los CaP de bajo riesgo ocupan el 39,1% de los diagnósticos en el grupo control y el 56,4% en el grupo de cribado [8]. La gestión adecuada de estos pacientes está en la base de la polémica sobre los beneficios y perjuicios del cribado CaP, puesto que el diagnóstico de un CaP con un bajo riesgo de progresión no va a beneficiar al paciente, pero sí puede perjudicarlo si es tratado con un prostatectomía radical o radioterapia, que pueden causar, entre otros efectos secundarios, incontinencia urinaria e impotencia.

En este sentido, se hace imprescindible comentar los resultados del estudio PIVOT (*Prostate Cancer Intervention versus Observation Trial*) publicados en 2017 tras efectuar un seguimiento de casi 20 años [25]. El estudio incluye una serie de 731 varones con CaP localizado que aleatoriamente fueron tratados con prostatectomía radical o bien seguidos mediante observación clínica. El estudio muestra que la cirugía no se asocia con una disminución de la mortalidad debida al CaP respecto a la observación, si bien tiene una mayor frecuencia de efectos adversos. Por otro lado, el estudio señala que la cirugía podría disminuir la mortalidad en pacientes con un tumor con un riesgo intermedio de recidiva, pero no en pacientes con tumores con bajo de recidiva.

Diferenciar entre pacientes con un bajo riesgo de progresión y pacientes que pueden beneficiarse de un tratamiento activo del tumor permitiría reducir los perjuicios inherentes al sobrediagnostico y sobretratamiento. La vigilancia activa ha sido propuesta como una herramienta útil para el manejo de pacientes con tumores con un bajo riesgo de progresión que nunca afectaran la vida del paciente [26]. Pacientes con un grado de Gleason menor a 7, una concentración de PSA inferior a 10 µg/L y poco material afecto por el tumor en la biopsia pueden ser incluidos en programas de vigilancia activa. Estos programas difieren la realización de un tratamiento activo a un futuro en que se detecte la progresión del tumor a través del seguimiento activo de los pacientes, que incluye la realización periódica de medidas de PSA y tacto rectal, así como sucesivas biopsias para valorar un aumento en el grado de Gleason.

La correcta clasificación de los tumores es clave para poder decidir con seguridad qué pacientes requieren un tratamiento radical y reservar la vigilancia activa únicamente para pacientes con CaP con un bajo riesgo de progresión. Las variables empleadas actualmente para seleccionar pacientes no están exentas de incertidumbre. Por un lado, debe considerarse que la obtención del grado de Gleason del tumor mediante biopsia tiene un cierto grado de incerteza, debido, entre otras razones, a la heterogeneidad del tumor y a la imprecisión del muestreo. También debemos tener en cuenta que la medida de PSA plantea diferencias de cuantificación en relación al ensayo empleado en su medida, mientras que las recomendaciones sugieren clasificar los pacientes teniendo en cuenta un único punto de corte de 10 µg/L que no tienen en cuenta las diferencias entre los sistemas de medida [27]. Igualmente, en pacientes en los cuales el CaP asienta en una próstata de gran volumen puede observarse una importante elevación de PSA que no se relaciona con la agresividad del tumor, sino con el volumen prostático, pero que en cambio descarta la inclusión del paciente en un protocolo de vigilancia activa. Por ello, algunos autores plantean que la densidad de PSA podría tener mayor utilidad para seleccionar pacientes para vigilancia activa que no el propio PSA [28]. Pese a todo los resultados obtenidos en los protocolos de vigilancia activa son positivos, con alrededor del 70% de los pacientes libres de tratamiento tras un seguimiento de 5 años y un riesgo de muerte por CaP a los 15 años de únicamente el 3% [29].

## Recomendaciones de las guías clínicas

El cribado del CaP sigue siendo un tema controvertido, en que deben valorarse para cada individuo las ventajas y riesgos que conlleva. Las guías de práctica clínica de las distintas sociedades reflejan este escenario, con recomendaciones, en ocasiones, contradictorias. La Tabla 1 describe un resumen de las recomendaciones de cinco sociedades relevantes [30], [31], [32], [33], [34] que, pese a las discrepancias que presentan, muestran una gran coincidencia en señalar la importancia de ofrecer a los pacientes una información suficiente para poder participar, de acuerdo a sus preferencias, en la toma de decisiones.

**Tabla 1:** Resumen de las recomendaciones de diversas guías clínicas sobre el cribado del CaP.

Guía de práctica clínica, año (referencia)	Recomendación general	Información adicional	Otros biomarcadores recomendados
EAU–ESTRO–SIOG, 2017 [30]	Ofrecer una estrategia individualizada adaptada al riesgo a varones bien informados con una esperanza de vida de al menos 10–15 años	Ofrecer la medida de PSA en hombres mayores de 50 años, mayores de 45 años con antecedentes familiares de CaP, afroamericanos mayores de 45 años, hombres con PSA > 1 µg/L a los 40 años o >2 µg/L a los 60 años	El porcentaje de PSA libre estratifica el riesgo de CaP cuando el PSA es de 4–10 µg/L y existe una biopsia previa negativa. Nuevos ensayos para la estratificación del riesgo, incluidos PHI y 4Kscore, están destinados a reducir la cantidad de biopsias innecesarias en hombres con un PSA entre 2 y 10 µg/L
AUA, 2018 [31]	Para los hombres de 55–69 años deben valorarse beneficios y riesgos asociados al cribado y al tratamiento El cribado no está recomendado en hombres menores de 54 años con riesgo medio de CaP, en mayores de 70 años o en hombres con una esperanza de vida menor a 10–15 años	La decisión debe ser individualizada para hombres menores de 55 años con un riesgo elevado de CaP, entre los cuales afroamericanos y hombres con antecedentes familiares de cáncer metastásico	Los derivados de PSA (densidad de PSA, intervalos de referencia específicos de edad), la cinética de PSA (velocidad y tiempo de duplicación), el porcentaje de PSA libre, proPSA y PCA3 deben considerarse pruebas secundarias con potencial utilidad para determinar la necesidad de una biopsia de próstata, o bien para decidir su repetición
USPSTF, 2018 [32]	La decisión debe ser individualizada para hombres de 55 a 69 años. Previamente deberán analizar conjuntamente con su médico los potenciales beneficios y riesgos asociados al cribado Se recomienda la no realización del cribado en hombres mayores de 70 años	No debe empezarse el cribado en individuos menores de 55 años con un riesgo medio de CaP ni tan solo con el objetivo de obtener un nivel base de PSA. Se reconoce un riesgo elevado de CaP en individuos con antecedentes familiares de CaP y en afroamericanos	No se recomiendan
NCCN, 2019 [33]	La participación en un programa de detección precoz de CaP debe realizarse tras recibir información apropiada sobre sus beneficios y riesgos. Los miembros del panel no han llegado a un acuerdo sobre a qué edad debe empezar y terminar el cribado del CaP	La mayoría de miembros del panel es favorable a iniciar el cribado a los 45 años. Para hombres entre 45 y 75 años el cribado se repetirá a intervalos de 2–4 años si el PSA es menor a 1 µg/L o cada 1-2 años si es superior a 1 µg/L	Un porcentaje de PSA libre <10%, PHI > 35 o 4Kscore (el cual estima la probabilidad de un CaP de alto riesgo) son potencialmente informativos sobre la decisión de realizar una biopsia Un índice de PCA3 > 35 es potencialmente informativo tras una biopsia negativa previa.
NICE, 2019 [34]	No debe ofrecerse automáticamente una biopsia de la próstata en función de un resultado de PSA Ofrecer resonancia magnética como test de primera línea a individuos con sospecha clínica de CaP localizado Los pacientes, debidamente informados, tienen derecho a participar en la toma de decisiones	En individuos con baja probabilidad de CaP según resonancia magnética y PSA elevado debe considerarse la densidad de PSA y la velocidad de PSA para decidir la realización de la biopsia, así como los antecedentes familiares de CaP	El índice de PCA3 y PHI no están recomendados para individuos estudiados por sospecha de CaP en los cuales la biopsia ha sido negativa

CaP, cáncer de próstata; *PCA3,* Prostate Cancer 3 gene; PHI, Prostate Health Index; PSA, antígeno específico de la próstata; AUA, American Urological Association; EAU, European Association of Urology; ESRO, European Society for Radiotherapy and Oncology; NCCN, National Comprehensive Cancer Network; NICE, National Institute for Health and Care Excellence; SIOG, International Society of Geriatric Oncology; USPSTF, US Preventive Services Task Force.

No hay acuerdo sobre en qué franja de edad debería plantearse el cribado. Según la *American Urological Association* (AUA) y la USPTF el cribado debería ser valorado entre los 55 y los 69 años para hombres con un riesgo medio de padecer CaP. En cambio, según la guía clínica que han redactado conjuntamente la *European Association of Urology* (EAU), la *European Society for Radiotherapy and Oncology* (ESRO) y la *International Society of Geriatric Oncology* (SIOG) debe ofrecerse, siempre que la esperanza de vida sea de 10–15 años, a partir de los 50 años y también en individuos más jóvenes en función de la concentración de PSA. La *National Comprehensive Cancer Network* (NCCN), si bien reconoce que no hay unanimidad entre los redactores de la guía, avanza la edad de inicio a los 45 años. Además tiene en cuenta la concentración de PSA para decidir de manera personalizada el intervalo de repetición de la medida de este biomarcador. La NCCN tampoco se pronuncia respecto a la edad a partir de la cual no es aconsejable la realización del cribado, mientras que otras guías (EAU–ESTRO–SIOG, AUA y USPSTF) señalan explícitamente que no se recomienda a partir de los 70 años o en individuos con una esperanza de vida menor a los 10–15 años.

Finalmente, en su última actualización, la guía del *National Institute for Health and Care Excellence* (NICE) no hace ningún comentario sobre el cribado y aconseja realizar una resonancia magnética en pacientes con sospecha clínica de CaP localizado. Asimismo, esta guía aconseja valorar la densidad de PSA y la velocidad de PSA en pacientes con baja probabilidad de CaP según el resultado de la resonancia magnética.

Nuevos biomarcadores, como el índice de PCA3, *prostate health index* (PHI) o proPSA y 4Kscore, son mencionados por su potencial utilidad en las guías de la EAU–ESTRO–SIOG, AUA y NCCN, pero, en cambio, no están recomendados ni por la guía de la USPTF ni por la de la NICE.

## Conclusiones

**Figura 1:**
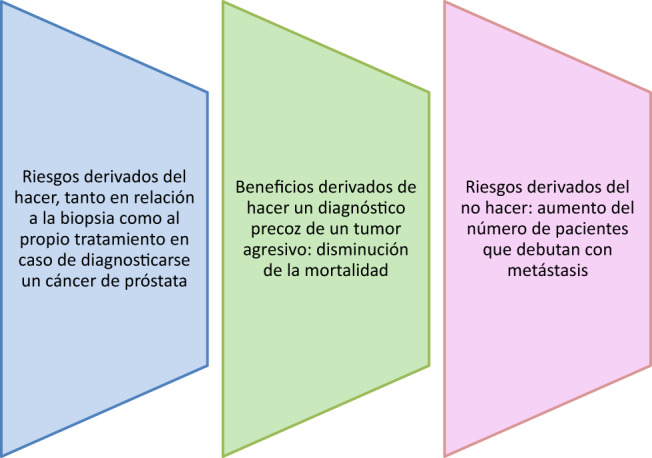
Balance de riesgos y beneficios en la detección precoz del cáncer de próstata.

El CaP es una enfermedad enormemente compleja, con grandes diferencias en su pronóstico, abarcando desde tumores indolentes que nunca van a afectar la vida del paciente hasta tumores letales resistentes a la castración. Esta heterogeneidad influye decisivamente en el balance entre beneficios y riesgos que se asocian a la práctica del cribado (Figura 1). Un diagnóstico precoz del tumor solo va a beneficiar a pacientes con tumores agresivos para los cuales dispongamos de un tratamiento efectivo que permita disminuir la mortalidad causada por el tumor. Por otro lado, deben considerarse los riesgos asociados al cribado, tanto por lo que respecta a la biopsia, que además en muchos casos será negativa dada la falta de especificidad del PSA, como al tratamiento en caso de que se detecte un CaP. Ello es especialmente relevante por cuanto un número importante de los CaP detectados tienen un bajo riesgo de progresión, por lo que el tratamiento puede ser innecesario. A este respecto los protocolos de vigilancia activa vienen a contrarrestar los efectos negativos de un tratamiento que en el caso de tumores indolentes es innecesario.

**Figura 2:**
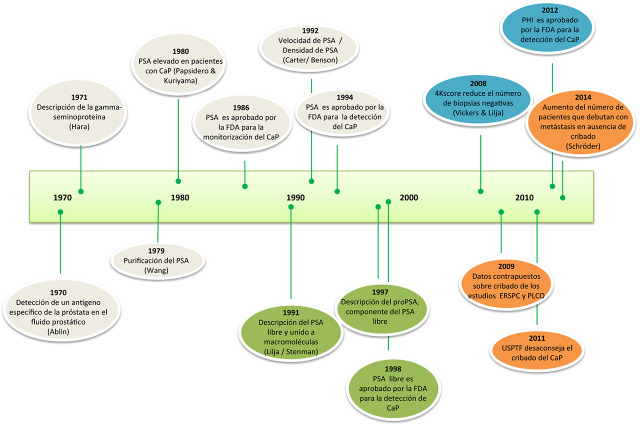
Cronograma de los biomarcadores empleados en la detección del CaP.

La disponibilidad de nuevos biomarcadores más específicos que el PSA y relacionados con la agresividad del tumor puede contribuir a mejorar los resultados obtenidos en los programas de cribado, disminuyendo por tanto sus desventajas. En los años 90 del siglo pasado se conocía que el PSA era una molécula constituida por una fracción libre y una fracción unida a diversas macromoléculas, principalmente la alfa-1-antiquimotripsina. Actualmente, se conocen muchas más fracciones, como el PSA intacto, el BPSA y el proPSA (Figura 2). El conocimiento de estas nuevas fracciones del PSA ha permitido que en el curso de los últimos años se hayan descrito nuevos biomarcadores, como PHI y 4Kscore. Ambos test, según los datos publicados, cumplen los objetivos de ser más específicos que el PSA y de relacionarse con la agresividad del tumor, facilitando la estratificación de los pacientes según su riesgo. Ello debería permitir su empleo para personalizar la necesidad de un tratamiento, distinguiendo con mayor certeza los pacientes con CaP indolente de aquellos con CaP agresivo.

PHI, que combina PSA total, PSA libre y la fracción-2 del proPSA (p2PSA), según la fórmula (p2PSA/PSA libre)*√PSA total, tiene un rendimiento superior que el PSA y que el porcentaje de PSA libre en la detección de CaP, con áreas bajo la curva entre 0,67 y 0,781 según los distintos estudios publicados [35]. Este test fue aprobado por la *Food and Drug Administration* (FDA) en junio del 2012 para decidir la realización de biopsia de la próstata en hombres de más de 50 años, PSA entre 4 y 10 µg/L, y un tacto rectal negativo. En nuestra experiencia, PHI se relaciona con el volumen de la próstata, habiendo obtenido áreas bajo la curva de 0,818, 0,716 y 0,654 para pacientes con un volumen prostático de ≤35, 36–50 y >50 cc, respectivamente [36].

El test 4Kscore es un índice que se calcula valorando PSA total, PSA libre, PSA intacto y la calicreína humana de tipo 2 junto con la edad del paciente, el tacto rectal y la existencia o no de una biopsia negativa previa. El objetivo del test, conocido también como test de 4 calicreínas, ya no es la detección del CaP, sino específicamente la detección del CaP de alto riesgo. Los resultados correspondientes a estudios retrospectivos realizados con este test muestran también una notable mejora con respecto a los obtenidos con el empleo de PSA, con áreas bajo la curva entre 0,798 y 0,903 [35]. Más recientemente, un estudio prospectivo confirmaba estos resultados, obteniendo un área bajo la curva de 0,821 [37].

Los estudios realizados sobre la rentabilidad de estos test muestran que su implementación, bien sea la de PHI [38], [39] o la de 4Kscore [40], mejoraría la relación coste-rendimiento del diagnóstico precoz del CaP. Como hemos mencionado anteriormente, algunas sociedades incluyen estos test en sus recomendaciones, si bien no hay total unanimidad al respecto.

En la valoración de beneficios y riesgos del cribado del CaP hay que considerar finalmente los riesgos asociados a la no práctica del cribado. De hecho, son numerosos los estudios que han observado un incremento del número de pacientes que debutan con metástasis cuando el cribado deja de practicarse [8], [16], con el correspondiente aumento del número de muertes causadas por CaP [17].

La utilidad del PSA en el cribado del CaP ha sido y sigue siendo objeto de una encendida polémica, que se pone de manifiesto en las distintas recomendaciones de las organizaciones científicas [41]. La controversia no se limita a la incertidumbre que rodea a la realización del cribado, sino también al intervalo de edad en que podría ser aplicado, a la posible definición de grupos de riesgo según un PSA basal, al intervalo que debería haber entre las mediciones de PSA o a la utilidad de otros biomarcadores que mejoren la aportación del PSA. En esta revisión hemos presentado de forma crítica y coincidiendo con otros grupos, subrayando las deficiencias metodológicas de algunos trabajos, los datos que hasta el momento han aportado los estudios de cribado. En ello coincidimos plenamente con el planteamiento de otros autores [42]. También hemos ofrecido en esta revisión una reflexión sobre el balance entre beneficios y riesgos que debe preceder a una decisión sobre la utilidad o no del PSA en el cribado de esta enfermedad. En esta reflexión juega un papel clave valorar no tan solo las desventajas del hacer, sino también las que conlleva el no hacer el cribado. En los últimos años, nuevas aportaciones subrayan la necesidad de personalizar la respuesta sobre la conveniencia o no del cribado. Eapen et al. [43] proponen dejar atrás la dicotomía entre cribar a toda la población o no cribar a nadie, en favor de un cribado inteligente. También, en un sentido semejante, se expresan Carlsson y Roobol [44], quienes apuestan por un cribado de nueva generación, subrayando el interés de los nuevos biomarcadores y de realizar una estratificación del riesgo en función de un PSA basal. Creemos que estas aportaciones, que advocan por un cribado personalizado, abren una interesante vía de reflexión que podría cambiar el balance entre beneficios y riesgos y hacer oportuno, en función del riesgo de cada individuo, el cribado del CaP.
